# Evaluating prevalence, risk factors, and diagnostic techniques for *Cryptosporidium* infection in goats and surrounding water sources

**DOI:** 10.3389/fvets.2024.1498682

**Published:** 2024-12-19

**Authors:** Manahil Rafiq, Naimat Ullah Khan, Imad Khan, Mansoor Ahmad, Aiman Bibi, Mourad Ben Said, Hanène Belkahia, Muhammad Tariq, Silwat Saeed, Mostafa A. Abdel-Maksoud, Mohamed A. El-Tayeb, Sabiha Fatima, Bushra Hafeez Kiani, Akram A. Alfuraydi, Farhad Badshah

**Affiliations:** ^1^Department of Zoology, Abdul Wali Khan University, Mardan, Pakistan; ^2^College of Veterinary Sciences and Animal Husbandry, Abdul Wali Khan University, Mardan, Pakistan; ^3^Department of Chemistry, Hazara University Mansehra, Mansehra, Pakistan; ^4^Department of Basic Sciences, Higher Institute of Biotechnology of Sidi Thabet, University of Manouba, Manouba, Tunisia; ^5^Laboratory of Microbiology, National School of Veterinary Medicine of Sidi Thabet, University of Manouba, Manouba, Tunisia; ^6^College of Animal Science and Technology, Nanjing Agricultural University, Nanjing, China; ^7^Institute of Animal Sciences, Chinese Academy of Agricultural Sciences, Beijing, China; ^8^Botany and Microbiology Department, College of Science, King Saud University, Riyadh, Saudi Arabia; ^9^Department of Clinical Laboratory Science, College of Applied Medical Sciences, King Saud University, Riyadh, Saudi Arabia; ^10^Department of Biology and Biotechnology, Worcester Polytechnic Institute, Worcester, MA, United States; ^11^Shenzhen Branch, Guangdong Laboratory of Lingnan Modern Agriculture, Key Laboratory of Livestock and Poultry Multi-Omics of MARA, Agricultural Genomics Institute at Shenzhen, Chinese Academy of Agricultural Sciences, Shenzhen, China; ^12^State Key Laboratory of Animal Biotech Breeding, Institute of Animal Sciences, Chinese Academy of Agricultural Sciences, Beijing, China

**Keywords:** cryptosporidiosis, *Cryptosporidium* oocysts, goats, water sources, risk factors, Pakistan

## Abstract

**Background:**

*Cryptosporidium* spp. are protozoan parasites that infect the gastrointestinal tract of various animals, including goats, and can also contaminate water sources, posing a significant public health risk. Detecting *Cryptosporidium* oocysts in fecal and water samples is critical for understanding the epidemiology of cryptosporidiosis and implementing appropriate control measures. Various staining methods, such as the Modified Ziehl-Neelsen (ZN) and Kenyon’s Acid-Fast (KAF) staining techniques, are employed to identify these oocysts. This study compared the effectiveness of these two staining methods in detecting *Cryptosporidium* oocysts in goat feces and water samples across different geographic regions in district of Mardan, Khyber Pakhtunkhwa, Pakistan, and other factors such as genders, age groups, diarrheal statuses, and feeding regimes.

**Methods:**

A total of 300 fecal and 300 water samples were collected from goats and water sources in four geographic regions: Tehsil Katlang, Tehsil Takhtbhai, Tehsil Mardan, and Tehsil Lundkhwarh. Samples were categorized based on gender, age group (<1 year, 1–2 years, and >2 years), diarrheal status, and feeding regime (stall feeding, grazing). The two staining methods, ZN and KAF staining, were employed to detect *Cryptosporidium* oocysts. The detection rates were calculated, and statistical analyses were performed to compare the effectiveness of the two methods across different categories.

**Results:**

The overall detection rates of *Cryptosporidium* oocysts for fecal samples were 61.00% (95% CI: 55.22–66.55%) using the ZN method and 63.33% (95% CI: 57.60–68.79%) using KAF method, with no significant difference (*p* > 0.05). The highest detection rate was observed in Tehsil Katlang (70.66%) with the ZN method and in both Tehsil Katlang and Takhtbhai (66.66%) with the KAF staining method, having no significant difference (*p* > 0.05). Gender-wise analysis in fecal samples showed similar detection rates for males and females, with no significant differences. Age-wise, the highest detection rates were found in the youngest age group (<1 year) using the ZN method, and in the oldest age group (>2 years) using KAF staining, with no significant differences between age groups. Diarrheal status analysis indicated higher detection rates in diarrheic goats for both methods, with the highest detection rate in the diarrheic group of Tehsil Katlang (84.61%) using the ZN method. Feeding regime analysis showed no significant differences between stall-fed and grazing goats. For water samples, the detection rates of *Cryptosporidium* oocysts were significantly different between the two methods. The ZN technique had a significantly higher overall detection rate of 16.00% (95% CI: 12.03–20.64%) compared to 1.00% (95% CI: 0.20–2.89%) for KAF staining (*p* < 0.001). The performance of the two staining methods for the detection of *Cryptosporidium* oocysts in contaminated water samples from different geographic regions was also presented.

**Conclusion:**

Both ZN and KAF staining methods are effective for detecting *Cryptosporidium* oocysts in goat feces. However, in water samples, the ZN method showed a significantly higher detection rate compared to KAF staining method, suggesting its suitability for environmental surveillance. These findings highlight the importance of integrating reliable diagnostic techniques with public health interventions to mitigate the zoonotic risks of cryptosporidiosis.

## 1 Introduction

*Cryptosporidium* is a significant enteric protozoan parasite that can infect a wide range of hosts, including humans, livestock, and wildlife (1). This apicomplexan parasite is the causative agent of cryptosporidiosis, a diarrheal disease that can be particularly severe in immunocompromised individuals (2). *Cryptosporidium* species have a complex life cycle, with both asexual and sexual stages, which allows them to effectively colonize and persist within the gastrointestinal tract of their hosts (3).

Cryptosporidial infections in goats can lead to diarrhea, weight loss, and reduced productivity, posing a threat to goat farming and the livelihoods of small-scale farmers (3, 4). Goats are considered important reservoirs of *Cryptosporidium*, as they can harbor both host-adapted and zoonotic species of the parasite (4, 5). Infected goats can shed large numbers of *Cryptosporidium* oocysts in their feces, which can contaminate the environment and serve as a source of infection for other animals and humans (1).

The prevalence of *Cryptosporidium* infection in goats can vary widely, depending on the geographical region, management practices, and other factors (6). Understanding the epidemiology of *Cryptosporidium* in goats, including the identification of risk factors associated with infection, is crucial for the development of effective control and prevention strategies (5, 7). Studies like Peng et al. (8) have shown how farm management practices, age, and health status significantly influence *Cryptosporidium* prevalence in yaks, emphasizing similar risk factors observed in goats. Additionally, molecular characterization studies such as Shehata et al. (9) demonstrate the value of using genotyping to identify zoonotic *Cryptosporidium* strains in livestock and their impact on host health. For instance, a recent meta-analysis highlighted that infections in livestock often reflect regional variations in climate and water management practices, making localized studies essential for effective parasite control (10).

The zoonotic potential of *Cryptosporidium* species poses a public health concern, as infected animals can serve as a reservoir for transmission to humans (1, 5). Similarly, other zoonotic pathogens, such as Toxoplasma gondii, have demonstrated significant flow within the food chain, necessitating robust surveillance measures in livestock (11). *Cryptosporidium* is considered an important zoonotic pathogen, with several species and genotypes capable of infecting both humans and animals (12, 13). Outbreaks of cryptosporidiosis have been linked to direct contact with infected livestock, as well as the consumption of contaminated water or food (6, 14). Recent research shows that children, elderly, and immunocompromised individuals are particularly vulnerable to severe cryptosporidiosis when exposed to contaminated water or animal environments (15).

Contaminated water sources, such as surface water, groundwater, and drinking water, can also be a source of *Cryptosporidium* transmission, contributing to the spread of the parasite in both human and animal populations (6, 16). Recent investigations, conducted by Hussain et al. (17), highlight the diversity of aquatic parasites in pristine water sources. They demonstrate that even minimally disturbed ecosystems can harbor a wide range of protozoan pathogens, including *Cryptosporidium*, which poses significant zoonotic risks. Water-borne cryptosporidiosis outbreaks have been reported worldwide, highlighting the importance of monitoring water quality for the presence of *Cryptosporidium* (18, 19). The environmental persistence and chlorine resistance of *Cryptosporidium* oocysts make them a significant challenge for water treatment and disinfection processes (7, 20). This resilience is emphasized in recent studies on waterborne outbreaks in low-resource areas, where limited water treatment facilities compound the risk of zoonotic transmission (21).

Cryptosporidiosis is an emerging zoonotic disease with significant implications for both human and animal health. Recent studies emphasize the role of livestock as primary reservoirs for *Cryptosporidium*, contributing to environmental contamination and waterborne transmission risks. For instance, research by Javed and Alkheraije (22), highlights the spread of *Cryptosporidium* via contaminated food and water, underscoring the importance of farm hygiene and water quality management in mitigating zoonotic risks. Studies conducted in diverse geographic settings illustrate the widespread nature of *Cryptosporidium* transmission across agricultural regions. A study from Mexico demonstrated substantial prevalence of *Cryptosporidium* in both domestic and wild animals, reinforcing the need for rigorous monitoring of animal and environmental contamination (23). Similarly, research on free-ranging livestock in Tibet found that shared water sources substantially heighten infection risks, illustrating how environmental factors can influence the spread of *Cryptosporidium* (24). These insights underscore the necessity of surveillance efforts, especially in agricultural regions where water contamination poses public health risks.

Accurate diagnosis and understanding the prevalence and risk factors associated with *Cryptosporidium* infections in goats and contaminated water sources are crucial for the development of effective control and prevention strategies (7). Robust diagnostic techniques, such as microscopy, immunoassays, and molecular methods, are necessary to accurately identify *Cryptosporidium* species and genotypes, which can have different host specificities and pathogenic potential (12, 25). Epidemiological studies investigating the prevalence and distribution of *Cryptosporidium* in goats and water sources can provide valuable insights into the transmission dynamics and risk factors associated with these infections (26, 27).

Limited research has been conducted on the epidemiology of *Cryptosporidium* infections in goats and the contamination of water sources in Pakistan (12, 13). Previous studies in the country have primarily focused on human cryptosporidiosis, with limited data available on the situation in livestock and the environment. This knowledge gap highlights the need for more comprehensive investigations into the prevalence and risk factors associated with *Cryptosporidium* infections in the Pakistani context, particularly in the important agricultural sector of goat farming.

This study represents the first comprehensive investigation in Pakistan to evaluate the prevalence of *Cryptosporidium* infection in goats within the Mardan district, Khyber Pakhtunkhwa. It aims to identify associated risk factors, detect *Cryptosporidium* in surrounding water sources, and assess the performance of diagnostic techniques, with a novel emphasis on comparing the efficacy of the Modified Ziehl-Neelsen (ZN) and Kenyon’s Acid-Fast (KNKAF) staining methods for detecting *Cryptosporidium* in both fecal and water samples.

## 2 Materials and methods

### 2.1 Sampling area

**Figure 1:**
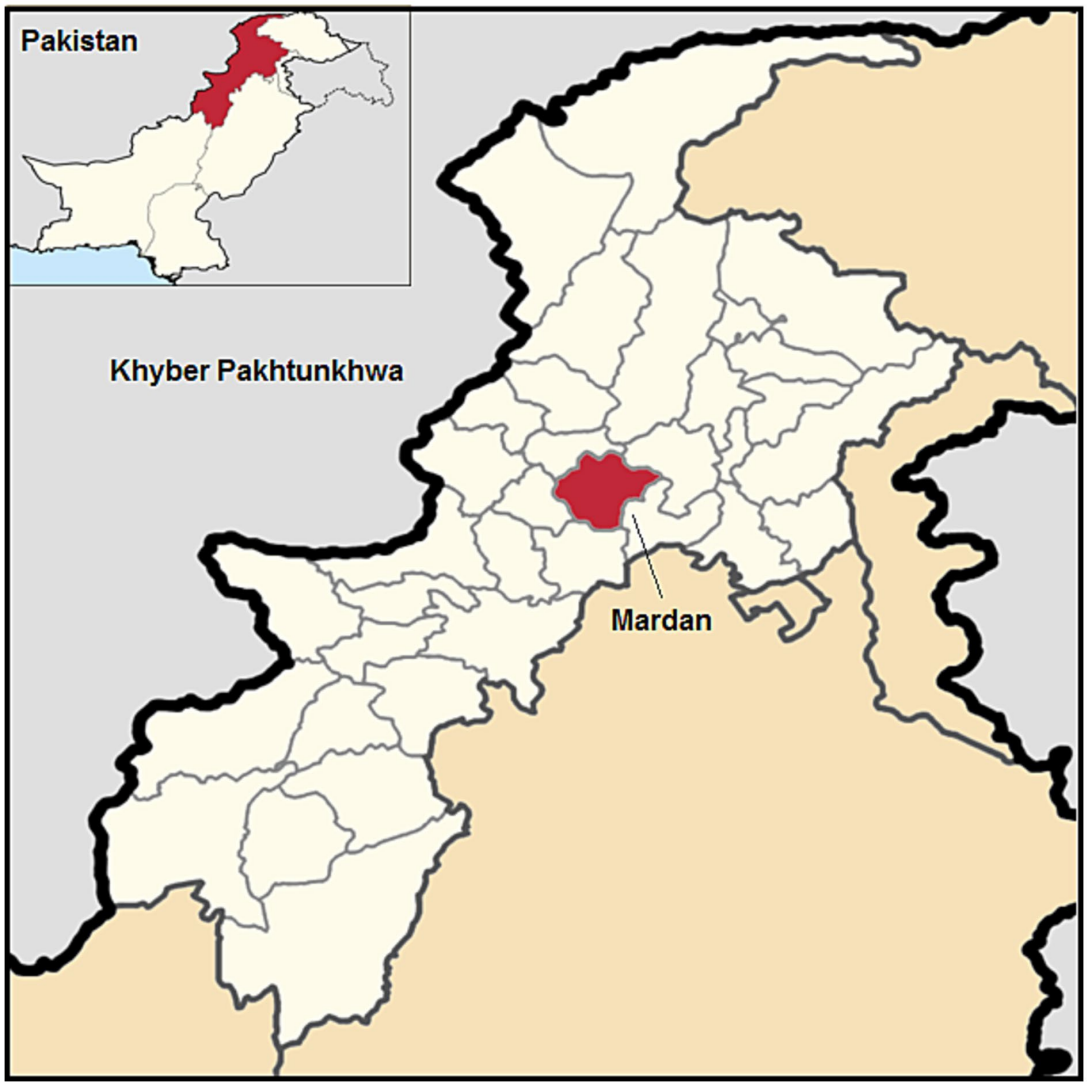
Map of the Mardan District, Khyber Pakhtunkhwa, Pakistan.

The study was conducted in the district of Mardan, located in KP, Pakistan, at coordinates 34.19860N and 72.04040E (Figure 1). Mardan is situated in the southwest of the district at an altitude of 283 meters (928 ft). The district comprises five different Tehsils, of which four were selected: Katlang, Takht Bahi, Mardan, and Lund-Khwarh. Within these Tehsils, five villages were randomly selected for sampling in each Tehsil. The coordinates and elevations of the selected Tehsils are as follows: Katlang (34°21′7.41″N, 72°4′35.2″E, 375.14 m), Takht Bahi (34°19′9.00″N, 71°56′26.99″E, 416 m), Mardan (34°12′7.02″N, 72°03′9.14″E, 337 m), and Lund-Khwarh (34°22′59.99”N, 71°58′59.99″E, 371 m). These locations were chosen to represent the diverse geographic and environmental conditions within the district.

### 2.2 Questionnaire

A detailed questionnaire was designed to systematically collect all relevant data related to the host and environmental conditions for this study on *Cryptosporidium* spp. in goats and water sources. Information such as age, geographic area, sex, feeding habits, and disease conditions, among other variables, were recorded prior to sample collection. This data collection was essential for understanding the epidemiology of cryptosporidiosis and implementing appropriate control measures. Participation of the goat owners was mandatory during the filling of the questionnaire to ensure the accuracy and completeness of the information. Owners provided detailed insights about each animal and water source, allowing for a thorough analysis of factors potentially influencing the prevalence of *Cryptosporidium* infections.

### 2.3 Sample collection, filtration, and preservation

#### 2.3.1 Fecal sample collection and preservation

A total of 300 fecal samples were randomly collected from goat farms and home-kept goats with different feeding habits, such as stall feeding and grazing. Samples were gathered from goats of various age groups: less than 1 year (G1), 1–2 years (G2), and 3–5 years (G3). All samples came from flocks owned by private owners. Each farm typically had about 200–250 goats, while home-kept goats ranged from 3 to 10 or more. About 5–10 grams of feces were collected in sterile plastic bottles using appropriate techniques and protective measures, including gloves, forceps, and water-resistant tags. Fecal samples were collected in two ways: from freshly passed feces and directly from the rectum. After collection, formalin was added to preserve the samples. Before further processing, the samples were stored in a refrigerator at −60°C.

#### 2.3.2 Collection and preservation of water samples

Water samples (*n* = 300) of approximately 100 ml were collected in sterilized plastic bottles from various sources, including surface water, groundwater (tubewells), wells, canals, and tap water. Samples were collected from a depth of about 20–30 cm below the surface. The samples were then filtered through gauze or filter cartridges with a pore size of 0.4–1 μm (28, 29). The filtered samples were stored in a refrigerator at 1–10°C and delivered to the laboratory within 96 h. For further analysis, including staining, a volume of 10 μl (one drop) was used (28, 30, 31).

### 2.4 *Cryptosporidium* oocytes identification

*Cryptosporidium* oocysts were detected in both freshwater and fecal samples using two diagnostic techniques: (i) Ziehl-Neelsen acid-fast staining (ZN) and (ii) Kenyon’s acid-fast staining (KAF) (32). Prepared slides were properly stained and examined under a microscope. Oocysts were identified based on their distinctive size, shape, and staining characteristics.

### 2.5 Prevalence calculation

The prevalence rate of *Cryptosporidium* in goat and water samples was calculated using the following formula:

Prevalence rate (%) = (total number of positive samples/total number of examined samples) × 100.

### 2.6 Statistical analysis

The collected data was analyzed using various software tools. Microsoft Excel was used to organize and store data for further analysis. Exact confidence intervals (CI) for prevalence rates at the 95% level were calculated. to compare differences in prevalence rates among various variables of each related risk factor, chi square test or Fisher’s exact test were performed using Epi Info 6.01 (CDC, Atlanta, USA) with a cut-off value of 0.05.

## 3 Results

### 3.1 *Cryptosporidium* oocyst detection in goat feces

Overall, the detection rate of *Cryptosporidium* oocysts was 61.00% (95% CI: 55.22–66.55%) for the ZN method and 63.33% (95% CI: 57.60–68.79%) for the KAF staining method (Table 1). The highest detection rate of oocysts by the ZN method was observed in the Tehsil Katlang region, reaching 70.66% (95% CI: 59.02–80.61%), while the lowest rate was recorded in the Tehsil Takhtbhai and Tehsil Lundkhwarh regions, with 56.00% (95% CI: 44.05–67.45%) in both cases. The statistical analysis revealed no significant difference between the two staining methods (*p* > 0.05). Regarding the KAF staining method, the highest rate was observed in the Tehsil Katlang and Tehsil Takhtbhai regions, with 66.66% (95% CI: 54.83–77.13%) in both cases, while the lowest rate was recorded in the Tehsil Lundkhwarh region, with 57.33% (95% CI: 45.37–68.69%; Table 1).

**Table 1:** Comparison of ZN and KAF methods for the Detection of *Cryptosporidium* oocysts in goat feces.

Geographic region	ZN staining		KAF staining	
	Positive/total, rate (%, C.I.^1^)	*p* value	Positive/total, rate (%, C.I.^1^)	*p* value
Tehsil Katlang	53/75 (70.66, 59.02–80.61)	0.210	50/75 (66.66, 54.83–77.13)	0.594
Tehsil Takhtbhai	42/75 (56.00, 44.05–67.45)		50/75 (66.66, 54.83–77.13)	
Tehsil Mardan	46/75 (61.33, 49.37–72.36)		47/75 (62.66, 50.73–73.56)	
Tehsil Lundkhwarh	42/75 (56.00, 44.05–67.45)		43/75 (57.33, 45.37–68.69)	
Total	183/300 (61.00, 55.22–66.55)		190/300 (63.33, 57.60–68.79)	

The statistical analysis revealed no significant difference between the two staining methods (*p* > 0.05).

1 C.I.: 95% confidence interval.

### 3.2 *Cryptosporidium* oocyst detection by gender across geographic regions

The total detection rates using the ZN staining method were 63.80% (95% CI: 53.85–72.96%) in males and 65.07% (95% CI: 56.07–73.35%) in female. For the KAF staining method, the total detection rates were 64.86% (95% CI: 55.22–73.68%) for males and 67.52% (95% CI: 58.24–75.88%) for females. For the ZN staining method, the highest detection rate was observed in the Tehsil Katlang region for both males (70.37, 95% CI: 49.81–86.24%) and females (69.69, 95% CI: 51.28–84.40%). The lowest rate was recorded in the Tehsil Lundkhwarh region for males (59.00, 95% CI: 36.35–79.29%) and in the Tehsil Takhtbhai region for females (54.83, 95% CI: 36.03–72.68%). The statistical analysis showed no significant difference in the detection rates between males and females across all regions (*p* > 0.05; Table 2). Regarding the KAF staining method, the highest detection rate was observed in the Tehsil Takhtbhai region for both males (70.83, 95% CI: 48.90–87.38%) and females (70.37, 95% CI: 49.81–86.24%). The lowest rate was recorded in the Tehsil Lundkhwarh region for males (60.71, 95% CI: 40.57–78.49%) and in the Tehsil Mardan region for females (69.23, 95% CI: 48.21–85.67%). Similarly, the statistical analysis revealed no significant difference in the detection rates between males and females across all regions (p > 0.05; Table 2).

**Table 2:** Comparison of ZN and KAF Staining for the detection of *Cryptosporidium* oocysts in goat feces by gender.

Geographic region	ZN staining			KAF staining		
	Positive/total, rate (% ± C.I.^1^)		*p* value	Positive/total, rate (% ± C.I.^1^)		*p* value
	Male	Female		Male	Female	
Tehsil Katlang	19/27 (70.37, 49.81–86.24)	23/33 (69.69, 51.28–84.40)	0.955	18/27 (66.66, 46.03–83.48)	18/26 (69.23, 48.21–85.67)	0.843
Tehsil Takhtbhai	18/30 (60.00, 40.60–77.34)	17/31 (54.83, 36.03–72.68)	0.881	17/24 (70.83, 48.90–87.38)	19/27 (70.37, 49.81–86.24)	0.971
Tehsil Mardan	17/26 (65.38, 44.33–82.78)	23/32 (71.87, 53.25–86.25)	0.805	20/32 (62.50, 43.69–78.89)	18/26 (69.23, 48.21–85.67)	0.795
Tehsil Lundkhwarh	13/22 (59.00, 36.35–79.29)	19/30 (63.33, 43.85–80.07)	0.982	17/28 (60.71, 40.57–78.49)	24/38 (63.15, 45.99–78.18)	0.840
Total	67/105 (63.80 ± 53.85–72.96)	82/126 (65.07, 56.07–73.35)	0.949	72/111 (64.86, 55.22–73.68)	79/117 (67.52, 58.24–75.88)	0.776

1 C.I.: 95% confidence interval.

### 3.3 *Cryptosporidium* oocysts detection in goat feces across age groups and geographic regions

For the ZN staining method, the highest detection rate was observed in the youngest age group (< 1 year), with 66.00% (95% CI: 55.84–75.17%). The lowest rate was seen in the oldest age group (> 2 years), with 57.00% (95% CI: 46.71–66.86%). However, the differences between the age groups were not statistically significant (*p* = 0.353). For KAF staining method, the highest detection rate was observed in the oldest age group (> 2 years), with 67.00% (95% CI: 56.88–76.08%). The lowest rate was seen in the middle age group (1–2 years), with 59.00% (95% CI: 48.71–68.73%). Again, the differences between the age groups were not statistically significant (*p* = 0.472). The highest detection rate by the ZN staining method was observed in age groups 1 and 2 of the Mardan tehsil, with a rate of 80.00% (95% CI: 59.29–93.16%). The lowest rate was observed in age group 3 of the Lundkhwarh tehsil, with 48.00% (95% CI: 27.79–68.69%). No statistically significant difference was observed between the age groups for this method (*p* = 0.353). For the Kenyon acid-fast staining method, the highest detection rate was observed in age group 3 of the Takhtbhai tehsil, with 80.00% (95% CI: 59.29–93.16%). The lowest rate was observed in age group 2 of the Takhtbhai tehsil, with 52.00% (95% CI: 31.30–72.20%). No statistically significant difference was observed between the age groups for this method (*p* = 0.472). In the Mardan tehsil, the detection rate of *Cryptosporidium* oocysts by the ZN staining method was the highest in age group 1, with 80.00% (95% CI: 59.29–93.16%). This rate decreased significantly in the higher age groups, reaching 64.00% (95% CI: 42.52–82.02%) in age group 2 and only 40.00% (95% CI: 21.12–61.33%) in age group 3 (Table 3). The statistical analysis revealed a significant difference between the age groups (*p* = 0.013), indicating that the age of the goats has a significant impact on the detection of *Cryptosporidium* oocysts by this staining method in the Mardan tehsil (Table 4).

**Table 3:** Comparison of ZN and KAF staining for the detection of *Cryptosporidium* oocysts in goat feces by age group.

Geographic region	ZN staining				KAF staining			
	Positive/total (Rate %, C.I.^1^)			*p* value	Positive/total (Rate %, C.I.^1^)			*p* value
	Age group 1	Age group 2	Age group 3		Age group 1	Age group 2	Age group 3	
Tehsil Katlang	20/25 (80.00, 59.29–93.16)	18/25 (72.00, 50.61–87.92)	15/25 (60.00, 38.66–78.87)	0.294	18/25 (72.00, 50.61–87.92)	17/25 (68.00, 46.49–85.05)	16/25 (64.00, 42.52–82.02)	0.832
Tehsil Takhtbhai	14/25 (56.00, 34.92–75.59)	14/25 (56.00, 34.92–75.59)	19/25 (76.00, 54.87–90.64)	0.240	17/25 (68.00, 46.49–85.05)	13/25 (52.00, 31.30–72.20)	20/25 (80.00, 59.29–93.16)	0.108
Tehsil Mardan	20/25 (80.00, 59.29–93.16)	16/25 (64.00, 42.52–82.02)	10/25 (40.00, 21.12–61.33)	0.013^*^	17/25 (68.00, 46.49–85.05)	13/25 (52.00, 31.30–72.20)	17/25 (68.00, 46.49–85.05)	0.401
Tehsil Lundkhwarh	12/25 (48.00, 27.79–68.69)	17/25 (68.00, 46.49–85.05)	13/25 (52.00, 31.30–72.20)	0.320	13/25 (52.00, 31.30–72.20)	16/25 (64.00, 42.52–82.02)	14/25 (56.00, 34.92–75.59)	0.682
Total	66/100 (66.00, 55.84–75.17)	65/100 (65.00, 54.81–74.27)	57/100 (57.00, 46.71–66.86)	0.353	65/100 (65.00, 54.81–74.27)	59/100 (59.00, 48.71–68.73)	67/100 (67.00, 56.88–76.08)	0.472

All samples were collected from different age groups, i.e., group 1 = < 1 year, group 2 = 1–2 years, and group 3= > 2 years.

1 C.I.: 95% confidence interval.

* Statistically significant test.

**Table 4:** Comparison of ZN and KAF Staining for the Detection of *Cryptosporidium* oocysts in goat feces by Diarrheal Status.

Geographic region	ZN staining			KAF staining		
	Positive/total, rate (% ± C.I.^1^)		*p* value	Positive/total, rate (% ± C.I.^1^)		*p* value
	Diarrheic	Non-diarrheic		Diarrheic	Non-diarrheic	
Tehsil Katlang	22/26 (84.61, 65.13–95.64)	31/49 (63.26, 48.28–76.57)	0.095	19/26 (73.07, 52.21–88.42)	31/49 (63.26, 48.28–76.57)	0.548
Tehsil Takhtbhai	6/11 (54.54, 23.37–83.25)	36/64 (56.25, 43.27–68.62)	0.916	6/11 (54.54, 23.37–83.25)	43/64 (93.47, 54.31–78.41)	0.637
Tehsil Mardan	4/6 (66.66, 22.27–95.67)	43/69 (62.31, 49.83–73.70)	0.833	5/6 (83.33, 35.87–99.57)	42/69 (60.86, 48.37–72.40)	0.514
Tehsil Lundkhwarh	4/6, (66.66, 22.27–95.67)	35/69 (50.72, 38.40–62.97)	0.746	4/6 (66.66, 22.27–95.67)	39/69 (56.52, 38.40–62.97)	0.746
Total	36/49 (73.46, 58.91–85.05)	145/251 (57.76, 51.39–63.95)	0.058	34/49 (69.38, 54.58–81.74)	155/251 (61.75, 55.43–67.79)	0.394

1 C.I.: 95% confidence interval.

### 3.4 Detection of *Cryptosporidium* oocysts by diarrheal status

The overall results show that the ZN staining method had a higher detection rate for *Cryptosporidium* oocysts in goat feces compared to KAF staining method. The total positive rate for the ZN method was 73.46% (95% CI: 58.91–85.05%) in the diarrheic group and 57.76% (95% CI: 51.39–63.95%) in the non-diarrheic group, with no statistically significant difference between the two groups (*p* = 0.058). For KAF staining method, the total positive rate was 69.38% (95% CI: 54.58–81.74%) in the diarrheic group and 61.75% (95% CI: 55.43–67.79%) in the non-diarrheic group, with no statistically significant difference between the two groups (*p* = 0.394). The highest detection rate for the ZN method was observed in the diarrheic group of the Katlang tehsil, with 84.61% (95% CI: 65.13–95.64%). The lowest rate for this method was seen in the non-diarrheic group of the Lundkhwarh tehsil, with 50.72% (95% CI: 38.40–62.97%). For KAF staining method, the highest detection rate was observed in the non-diarrheic group of the Takhtbhai tehsil, with 93.47% (95% CI: 54.31–78.41%). The lowest rate for this method was seen in the diarrheic group of the Takhtbhai tehsil, with 54.54% (95% CI: 23.37–83.25%; Table 4).

### 3.5 Detection of *Cryptosporidium* oocysts by feeding regime

Overall, the results show that the ZN staining method has a higher detection rate for *Cryptosporidium* oocysts in goat feces compared to KAF staining, particularly in the stall-feeding group. However, the differences between the feeding regimes were not statistically significant for the majority of the tehsils. For the ZN staining method, the highest detection rate was observed in the Tehsil Mardan region among goats under stall feeding, with a 100% (95% CI: 84.56–100%) positive rate. This was significantly higher than the detection rate in the grazing group of the same tehsil, which was 63.33% (95% CI: 43.85–80.07%; *p* = 0.004). In contrast, the lowest detection rate for the ZN method was seen in the grazing group of the Tehsil Lundkhwarh, with a rate of 44.44% (95% CI: 21.53–69.24%). The detection rate in the stall-feeding group of the same tehsil was higher at 64.28% (95% CI: 35.13–87.24%), but the difference was not statistically significant (*p* = 0.448). For KAF staining method, the highest detection rate was observed in the grazing group of the Tehsil Katlang, with a rate of 72.00% (95% CI: 50.61–87.92%). The lowest rate for this method was seen in the stall-feeding group of the Tehsil Lundkhwarh, with a rate of 55.55% (95% CI: 35.32–74.52%). However, the differences in detection rates between the feeding regimes were not statistically significant for any of the tehsils (*p* > 0.05; Table 5).

**Table 5:** Comparison of ZN and KAF staining for the detection of *Cryptosporidium* oocysts in goat feces by feeding regime.

Geographic region	ZN staining			KAF staining		
	Positive/total, rate (% ± C.I.^1^)		*p* value	Positive/total, rate (% ± C.I.^1^)		*p* value
	Stall feeding	Grazing		Stall feeding	Grazing	
Tehsil Katlang	15/22 (68.18, 45.12–86.13)	19/25 (76.00, 54.87–90.64)	0.786	12/20 (60.00, 36.05–80.88)	18/25 (72.00, 50.61–87.92)	0.595
Tehsil Takhtbhai	17/29 (58.62, 38.93–76.47)	13/22 (59.09, 36.35–79.29)	0.973	17/25 (68, 46.49–85.05)	15/23 (65.21, 42.73–83.62)	0.839
Tehsil Mardan	22/22 (100, 84.56–100)	19/30 (63.33, 43.85–80.07)	0.004^*^	16/27 (59.25, 38.79–77.61)	19/28 (67.85, 47.64–84.12)	0.702
Tehsil Lundkhwarh	9/14 (64.28, 35.13–87.24)	8/18 (44.44, 21.53–69.24)	0.448	15/27 (55.55, 35.32–74.52)	13/19 (68.42, 43.44–87.42)	0.566
Total	63/87 (72.41, 61.78–81.45)	59/95 (62.10, 51.57–71.86)	0.186	60/99 (60.60, 50.27–70.28)	65/95 (72.63, 58.07–77.57)	0.323

1 C.I.: 95% confidence interval.

* Statistically significant test.

### 3.6 Detection of *Cryptosporidium* oocysts in contaminated water samples by geographic region

When comparing the two staining methods, the ZN technique had a significantly higher overall detection rate of 16.00% (95% CI: 12.03–20.64%) compared to 1.00% (95% CI: 0.20–2.89%) for KAF staining (*p* < 0.001). For the ZN staining method, the highest detection rate was observed in Tehsil Katlang, with a positive rate of 24.00% (95% CI: 14.88–35.25%), which was not significantly different from the detection rates in the other tehsils (*p* > 0.05). The lowest detection rate for this method was observed in Tehsil Lundkhwarh, with a positive rate of 9.33% (95% CI: 3.83–18.28%). In contrast, KAF staining method showed much lower detection rates overall. The highest rate was observed in Tehsil Katlang, with a positive rate of 2.66% (95% CI: 0.32–9.30%), which was not significantly different from the other tehsils (*p* > 0.05). The Tehsil Takhtbhai and Tehsil Mardan had the lowest detection rates, with 0% positive samples (Table 6).

**Table 6:** Comparison of ZN and KAF staining for the detection of *Cryptosporidium* oocysts in water samples.

Geographic region	ZN staining		KAF staining	
	Positive/total, rate (% ± C.I.^1^)	*p* value	Positive/total, rate (% ± C.I.^1^)	*p* value
Tehsil Katlang	18/75 (24.00, 14.88–35.25)	0.087	2/75 (2.66, 0.32–9.30)	0.295
Tehsil Takhtbhai	13/75 (17.33, 9.56–27.81)		0/75 (0)	
Tehsil Mardan	10/75 (13.33, 6.58–23.15)		0/75 (0)	
Tehsil Lundkhwarh	7/75 (9.33, 3.83–18.28)		1/75 (1.33, 0.03–7.20)	
Total	48/300 (16.00, 12.03–20.64)		3/300 (1.00, 0.20–2.89)	

1 C.I.: 95% confidence interval.

### 3.7 Detection of *Cryptosporidium* oocysts in different water sources

Overall, the ZN staining method showed a significantly higher detection rate of 33.33% (95% CI: 23.05–44.91%) compared to 7.69% (95% CI: 2.87–15.99%) for the KAF staining method (*p* = 0.006). The ZN staining method showed the highest detection rate of 51.51% (95% CI: 33.54–69.20%) in stream water samples from the Tehsil Takhtbhai region, which was significantly higher than the detection rates in other water sources and regions (*p* < 0.001). In contrast, the ZN method had the lowest detection rate of 0% in well and tubewell water samples from the Tehsil Lundkhwarh and Tehsil Mardan regions. For the KAF staining method, the highest detection rate was 16.66% (95% CI: 2.08–48.41%) in stream water samples from the Tehsil Katlang region, which was not significantly different from the other water sources and regions (*p* = 0.081). The KAF staining method had the lowest detection rate of 0% in well and tubewell water samples from the Tehsil Lundkhwarh and Tehsil Mardan regions (Table 7).

**Table 7:** Comparison of ZN and KAF staining for the detection of *Cryptosporidium* oocysts in different water sources.

Geographic region	ZN staining					KAF staining				
	Positive/total, rate (% ± C.I.^1^)				*p* value	Positive/total, rate (% ± C.I.^1^)				*p* value
	Well	Tubewell	Stream	River		Well	Tubewell	Stream	River	
Tehsil Katlang	1/19 (5.26, 0.13–26.02)	3/23 (13.04, 2.77–33.58)	6/12 (50.00, 21.09–78.90)	9/20 (45.00, 23.05–68.47)	0.003^*^	0/19 (0)	0/23 (0)	2/12 (16.66, 2.08–48.41)	1/20 (5.00, 0.12–24.87)	0.081
Tehsil Takhtbhai	1/23 (4.34, 0.11–21.94)	1/35 (2.87, 0.07–14.91)	5/6 (83.33, 35.87–99.57)	6/12 (50.00, 2.09–78.90)	0.000^*^	2/23 (8.69, 1.07–28.03)	1/35 (2.85, 0.07–14.91)	2/6 (33.33, 4.32–77.72)	2/12 (16.66, 2.08–48.41)	0.083
Tehsil Mardan	0/34 (0)	1/19 (5.26, 0.13–26.02)	3/8 (37.50, 8.52–75.51)	6/15 (40.00, 16.33–67.71)	0.000^*^	1/34 (2.94, 0.07–15.32)	0/19 (0)	1/8 (12.5, 0.31–52.65)	2/15 (13.33, 1.65–40.46)	0.238
Tehsil Lundkhwarh	0/14 (0)	0/22 (0)	3/7 (42.85, 9.89–81.59)	5/31 (16.12, 5.45–33.72)	0.005^*^	0/14 (0)	0/22 (0)	0/7 (0)	1/31 (3.22, 0.08–16.70)	0.704
Total	2/90 (2.22, 0.27–7.79)	5/99 (5.05, 1.65–11.39)	17/33 (51.51, 33.54–69.20)	26/78 (33.33, 23.05–44.91)	0.000^*^	3/90 (3.33, 0.69–9.43)	1/99 (1.01, 0.02–5.49)	5/33 (15.15, 5.10–31.89)	6/78 (7.69, 2.87–15.99)	0.006^*^

1 C.I.: 95% confidence interval.

* Statistically significant test.

## 4 Discussion

In this study, we examined *Cryptosporidium* spp. infection in goats as well as in different water sources in four tehsils of Mardan district, Pakistan. Several risk factors such as age, sex, personal hygiene, poor sanitation system, diarrheic and non-diarrheic conditions, and the use of various water sources for drinking were studied. These factors may increase the risk of outbreaks worldwide or be a major cause of cryptosporidiosis, often neglected (33, 34). In our current study, fecal samples from symptomatic and asymptomatic goats were collected and examined for the presence of the suspected parasite.

The overall detection rates of *Cryptosporidium* oocysts were similar between the two methods, with 61.00% for the ZN method and 63.33% for the KAF staining method. No statistically significant difference was observed between the two staining methods, suggesting that both methods are equally effective for the detection of *Cryptosporidium* oocysts in goat feces. The consistency in the performance of these two methods indicates that they can be used interchangeably for the diagnosis of *Cryptosporidium* infection in goats. The high detection rates observed with both staining methods suggest a significant prevalence of *Cryptosporidium* infection in the goat population within the study area. The findings highlight the importance of implementing effective diagnostic techniques and control measures to reduce the impact of *Cryptosporidium* infection in goats, which can have implications for animal health, productivity, and potentially human health through zoonotic transmission (34). Additionally, advancements in vaccination strategies against zoonotic pathogens like Toxoplasma gondii provide a framework for improving livestock health and mitigating zoonotic risks (35). Similarly, a study conducted by Nemat et al. (36) on diarrheic sheep in Pakistan found that ZN and modified Ziehl-Neelsen (mZN) staining methods yielded comparable detection rates, indicating high prevalence levels in livestock. The consistency across these methods highlights their reliability as practical diagnostic tools in resource-limited settings. Our findings also emphasize the high prevalence of *Cryptosporidium* in the goat population in Mardan, supporting the need for effective diagnostic practices to manage infection and mitigate zoonotic transmission risks in regions with close human-animal contact.

The ZN method showed the highest detection rate (70.66%) in the Tehsil Katlang region, while the lowest rates (56.00%) were observed in the Tehsil Takhtbhai and Tehsil Lundkhwarh regions. For the KAF staining method, the highest detection rates (66.66%) were found in the Tehsil Katlang and Tehsil Takhtbhai regions, while the lowest rate (57.33%) was recorded in the Tehsil Lundkhwarh region. These geographical variations in the detection rates of *Cryptosporidium* oocysts may be influenced by factors such as climate, management practices, or other environmental conditions in the different regions (37).

Our data shows that the prevalence of cryptosporidiosis is generally higher in females than in males. In this study, the infection rate in females was 62.87% on ZN staining and 63.47% on KAF staining, compared to 58.01% on ZN and 62.59% on KAF in males. However, another study conducted in Iraq reported a higher infection rate in males (88.4%) than in females (85.5%), with no statistically significant difference (*p* > 0.05) (13). In contrast a study from Kirkuk province showed a higher infection rate in females (63.35%) than in males (58.75%) in Irak (7).

The prevalence of *Cryptosporidium* spp. infection in goats varies across different age groups. Age-wise, the highest detection rates were found in the youngest age group (<1 year) using the ZN method, and in the oldest age group (>2 years) using KAF staining, with no significant differences between age groups. These findings are consistent with other studies. For instance, a study from Pakistan reported that *Cryptosporidium* was most prevalent in goats aged 1 year (20.46%), followed by 1–2 years (13.73%) and 2–3 years (8.27%) (38). Similarly, a study from Serbia found that kids aged 5–21 days had higher mortality (40–50%) and morbidity (75–100%) rates due to *Cryptosporidium* infection compared to older animals (10). Further, a study in China reported an average *Cryptosporidium* prevalence of 2.23% in goats (16), while a Korean study observed the highest prevalence in goats under 2 months of age (5). In children, a study from Kirkuk Province, Iraq found the highest prevalence of *Cryptosporidium* infection in those under 1 and 2 years of age (32.35 and 26.24%, respectively) compared to older children (7). Additionally, *Cryptosporidium* infection has been reported in pre-weaned calves, with one study in Kuwait finding a prevalence of 33.1% in calves less than 1 month old with poor hygiene (18). Differences in detection across age groups could be attributed to immunological maturity and exposure levels. Younger goats might shed more oocysts detectable by ZN, while KAF may better visualize mature oocysts in older goats. These findings highlight the importance of age as a risk factor for *Cryptosporidium* infection in small ruminants and calves, with younger animals generally more susceptible to the parasite.

Our study found that the total prevalence of *Cryptosporidium* spp. in diarrheic goats in the Mardan district was 73.46% when using the ZN staining method. The prevalence in non-diarrheic goats was lower at 57.76%. When using the Kenyon acid-fast (KAF) staining method, the prevalence in diarrheic goats was 69.38%, while in non-diarrheic goats it was 61.75%. These findings align with other studies that have reported *Cryptosporidium* as a significant pathogen causing neonatal diarrhea in small ruminants such as goats, sheep, and calves. For instance, a study by Ali et al. (31) found that diarrhea, after malaria, is the second most crucial disease in livestock, accounting for 17% of cases. They documented a prevalence of 37.74% in non-diarrheic kids and 62.26% in diarrheic kids. Additionally, a study in Algeria by Bennadji et al. (25) reported a *Cryptosporidium* spp. infection rate of 25% in diarrheic goats and sheep. These results highlight the importance of *Cryptosporidium* as a major contributor to diarrheal disease in young small ruminants. The higher prevalence observed in diarrheic animals compared to non-diarrheic ones underscores the pathogenic role of this parasite in causing clinical disease.

Using KAF staining, the prevalence in grazing goats was 72.63%, while in stall-fed goats it was 60.60%. Using the ZN staining, the prevalence in grazing goats was 62.10%, compared to 72.41% in stall-fed goats. These findings align with several recent studies that have reported *Cryptosporidium* infections in children associated with different feeding practices. For instance, the prevalence of *Cryptosporidium* in stool samples was 16.93% in breastfed children, 59.63% in children who drank sterile/mineral water, and 35.79% in those who drank water from tanks, compared to only 4.58% in children using municipal water (7). In the present study, the researchers used both mZN and Kinyoun’s acid-fast stains to detect *Cryptosporidium* spp. in stool samples collected from goats in the Mardan district. They also detected *Cryptosporidium* in various water samples from the same locality, which may have been used by the host animals. Similar staining techniques, such as the ZN stain, have been used by other researchers for the detection of *Cryptosporidium* in stool samples (7, 12). Felefel et al. from Iraq reported *Cryptosporidium* spp. infection rate of 26.66% using the ZN stain, while the frequency was different when using other diagnostic methods, such as ELISA and PCR (12). These findings highlight the importance of considering management practices, such as grazing versus stall-feeding, in the epidemiology and control of *Cryptosporidium* infections in small ruminants. Water is an essential nutrient and a basic component of the diet, with no calories but important structural roles in cells. It is therefore crucial to investigate the influence of contaminated water in the transmission of *Cryptosporidium*, a zoonotic pathogen, to different animals. Ensuring the safety and security of drinking water is important for public health, and water used in farming raw vegetables can also be an indirect route of transmission for waterborne protozoa. Surveillance of various water sources in the study area revealed that, for the ZN staining method, detection rates varied significantly across water sources: 51.51% in streams, 33.33% in rivers, 5.05% in tubewells, and 2.22% in wells (*p* < 0.001). For Kenyon’s acid-fast staining, detection rates were 15.15% in streams, 7.69% in rivers, 3.33% in wells, and 1.01% in tubewells (*p* = 0.006). These findings align with a previous study by Abbas et al. from Pakistan (26), which reported an overall *Cryptosporidium* prevalence of 11.5% in water bodies, raw vegetables, and soil. The study emphasized that the frequency of *Cryptosporidium* was highest in sewage water (13%), followed by municipal water (10%) and canal water (9.5%). They highlighted that poor hygiene is a significant risk factor affecting the prevalence of *Cryptosporidium* and other parasites. Another study conducted in Igdir, Turkey, on water samples collected from springs found the prevalence of *Cryptosporidium* to be 10.1% by direct examination, 11.6% by nested PCR, and 7.2% by modified acid-fast staining (27). The study did not observe any significant differences in the physicochemical parameters of the water samples, such as pH, salinity, altitude, pressure, and temperature. These results highlight the importance of monitoring water sources and addressing issues related to water contamination and hygiene practices to reduce the transmission of *Cryptosporidium* and other waterborne pathogens to both animals and humans (39).

## 5 Conclusion

The findings from this study on evaluating the prevalence, risk factors, and diagnostic techniques for *Cryptosporidium* infection in goats and contaminated water sources underscores the critical need for integrated approaches to address this significant public health challenge. The high prevalence of *Cryptosporidium* detected in goat samples and the occurrence of this parasite in water samples highlights the zoonotic nature of this pathogen and the role of contaminated water sources in its transmission. Implementing effective surveillance, water quality monitoring, and targeted interventions to improve hygiene and sanitation practices are essential to reduce the spread of *Cryptosporidium* among animal and human populations. To enhance these efforts, we recommend adopting ZN staining as a reliable method for water surveillance and combining it with targeted educational campaigns for farmers to promote awareness and prevention of *Cryptosporidium* infections. Additionally, future studies should incorporate molecular diagnostic methods to complement staining techniques, enabling more precise identification and characterization of *Cryptosporidium* species. By adopting a One Health perspective that considers the interconnections between animal, human, and environmental health, researchers and public health authorities can work collaboratively to develop comprehensive strategies to reduce the burden of cryptosporidiosis. Continued research to optimize diagnostic techniques and inform evidence-based risk assessment and management will be crucial in safeguarding the health and well-being of both goats and human communities reliant on shared water resources.

## Data Availability

The raw data supporting the conclusions of this article will be made available by the authors, without undue reservation.
